# *De novo* transcriptome assembly and gene annotation for the toxic dinoflagellate *Dinophysis*

**DOI:** 10.1038/s41597-023-02250-8

**Published:** 2023-06-02

**Authors:** Chetan C. Gaonkar, Lisa Campbell

**Affiliations:** 1 Department of Oceanography, Texas A&M University, College Station, US; 2 Department of Biology, Texas A&M University, College Station, US

**Keywords:** Marine biology, Ecological genetics

## Abstract

Species within the dinoflagellate genus *Dinophysis* can produce okadiac acid and dinophysistoxins leading to diarrhetic shellfish poisoning. Since the first report of *D. ovum* from the Gulf of Mexico in 2008, reports of other *Dinophysis* species across US have increased. Members of the *D*. cf. *acuminata* complex (*D. acuminata*, *D. acuta*, *D. ovum*, *D. sacculus*) are difficult to differentiate due to their morphological similarities. *Dinophysis* feeds on and steals the chloroplasts from the ciliate, *Mesodinium rubrum*, which in turn has fed on and captured the chloroplasts of its prey, the cryptophyte *Teleaulax amphioxeia*. The objective of this study was to generate *de novo* transcriptomes for new isolates of these mixotrophic organisms. The transcriptomes obtained will serve as a reference for future experiments to assess the effect of different abiotic and biotic conditions and will also provide a useful resource for screening potential marker genes to differentiate among the closely related species within the *D*. cf. *acuminata*-complex. The complete comprehensive detailed workflow and links to obtain the transcriptome data are provided.

## Background & Summary

Diarrhetic Shellfish Poisoning (DSP) is a human illness caused by consumption of shellfish contaminated with okadaic acid and/or dinophysistoxins. The organisms responsible for producing these toxins include species in the marine dinoflagellate genus *Dinophysis*. Although a total of 137 *Dinophysis* species are taxonomically accepted, only 10 are known to produce DSP when humans consume filter-feeding shellfish that have concentrated these species^1,2^. An unusual feature of *Dinophysis* is that they are mixotrophic—that is, they rely on both photosynthesis and prey capture. They accomplish this by feeding on and stealing the chloroplasts from the ciliate, *Mesodinium rubrum*, which in turn has fed on and captured the chloroplasts of its prey, the cryptophyte *Teleaulax amphioxeia*. Many single-celled plankton are now recognized as mixotrophs^3^.

Until recently, DSP-related shellfish closures were reported primarily in Asian and European waters. The first incidence of *Dinophysis* occurrence at bloom levels in US was reported in 2008 for the Texas coast and lead to the closure of shellfish harvesting^4,5^. In the past decade, *Dinophysis* blooms have increased in frequency nationwide, so all coasts in the US now face closures of shellfish industries, but each event is linked to a different *Dinophysis* species. In the Gulf of Mexico, DSP and shellfish closures have been attributed to *D. ovum*^4^. Shellfish harvesting closures have been linked to blooms of *D. acuminata* and *D. fortii* in Puget Sound, WA^6^, to *D. acuminata* in Massachusetts^7^, and to *D. norvegica* in Maine^8^. Multiple species of toxigenic *Dinophysis* are present in the Chesapeake Bay^9^. Because of the morphological and genetic similarity of *D. acuminata* and *D. ovum*, counts of these two–along with *D. sacculus* and *D. acuta*—are often lumped together as “*D*. cf. *acuminata*-complex” in monitoring programs utilizing light microscopy^9^. Recent studies, however, have shown that *D. acuminata* and *D. ovum* have unique toxin profiles^10^. The diversity of *Dinophysis* species and toxigenicity in different regions of the US suggests that effective management will require examination of the environmental factors that influence their growth.

**Fig. 1:**
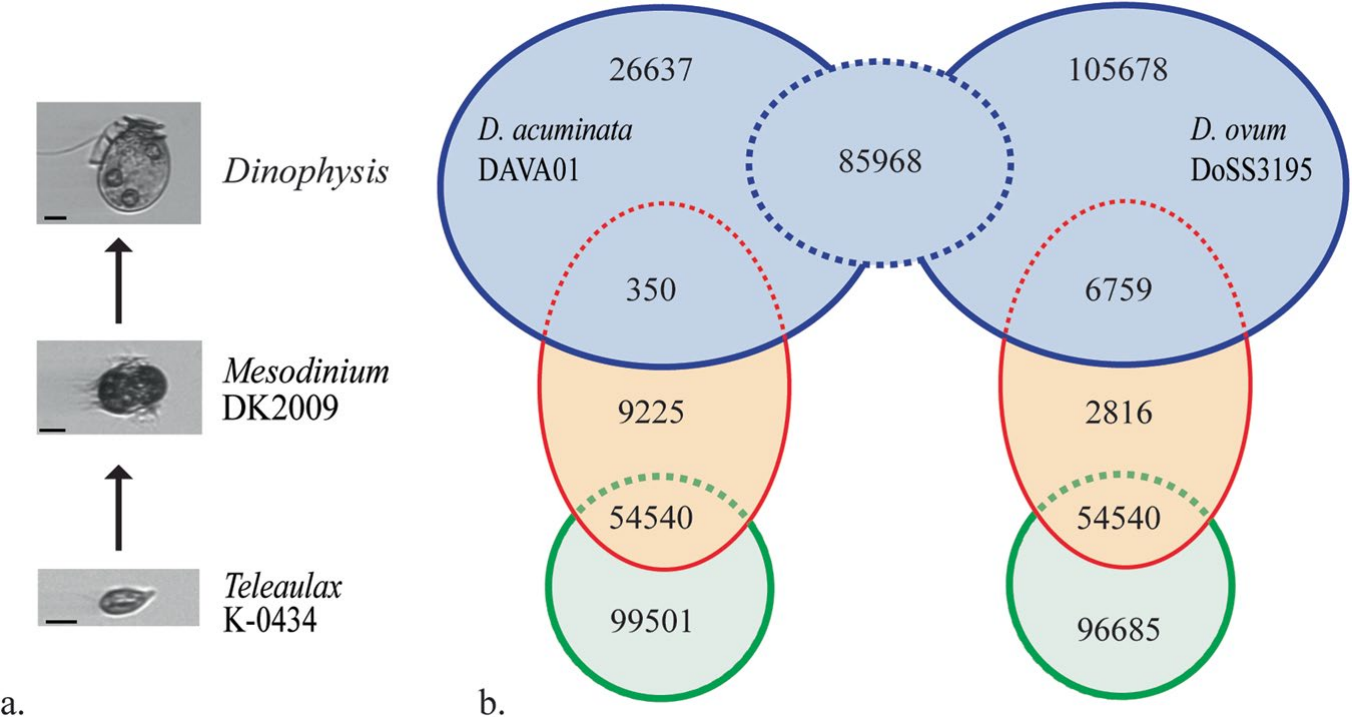
(**a**) The food chain supporting the mixotrophic dinoflagellate *Dinophysis*, includes *Mesodinium rubrum* and *Teleaulax amphioxeia*^39^. Images from the Imaging FlowCytobot in the Gulf of Mexico, Texas coast^4^. Scale bar = 10 μm. (**b**) Venn diagrams showing the unique transcripts for each organism, with the shared transcripts shown in the overlapping areas. Note that the larger number of transcripts discovered for *D. ovum* was due to the higher sequencing depth, so the number of shared transcripts between *D. ovum* and *M. rubrum* also was higher.

**Fig. 2:**
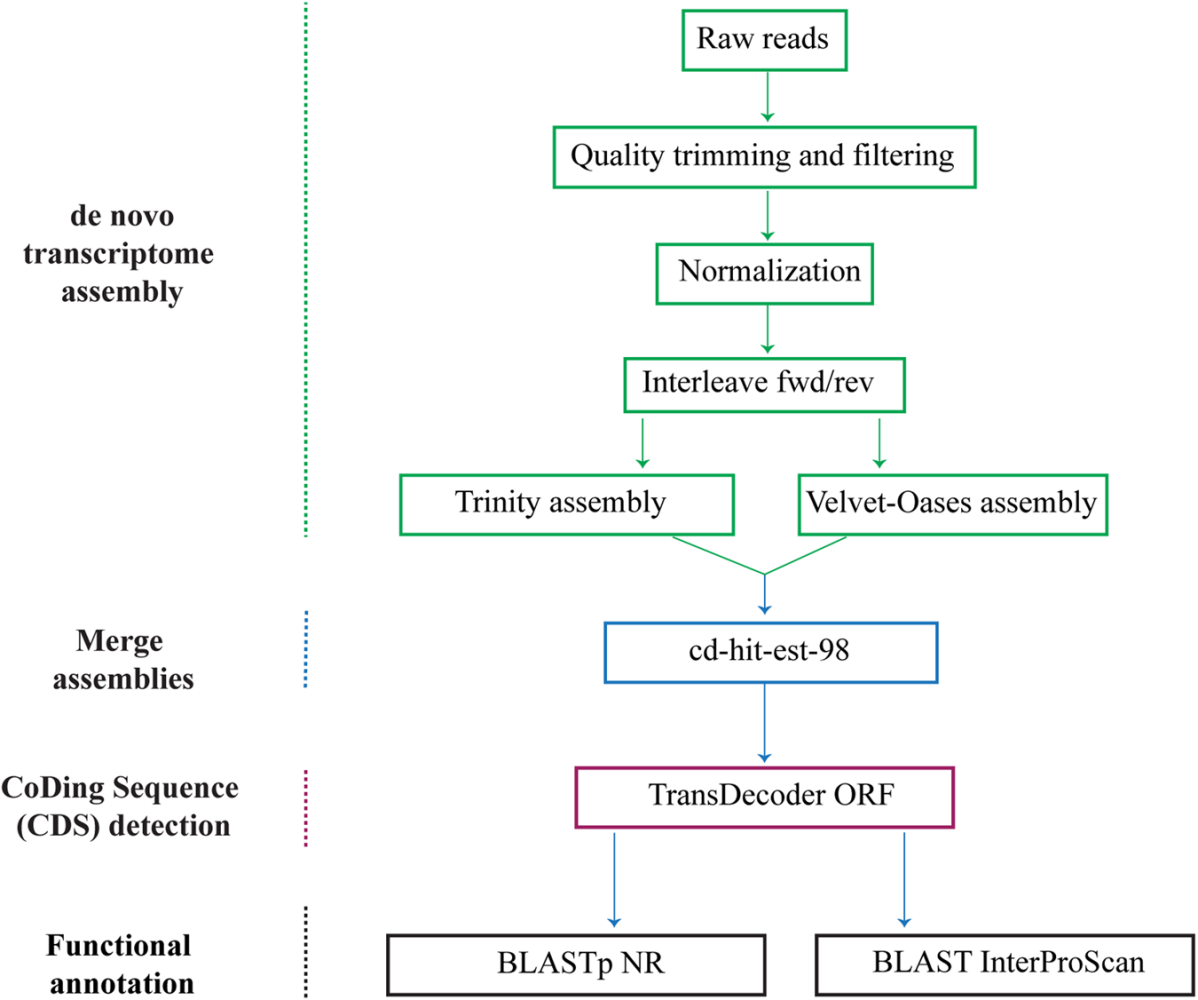
The bioinformatics tools used for assembly of the non-model organisms *Dinophysis, Mesodinium*, and *Teleaulax*. Quality trimming and filtering were accomplished with BBmap (https://sourceforge.net/projects/bbmap/) and SortMeRNA^16^, followed by normalization with the BBnorm function and interleaving the forward reads (fwd) and reverse reads (rev) using the BBrepair function in the BBMap package. Assemblies were generated with Trinity^17^ and Velvet-Oases^18,19^ and merged with cd-hit-est at 98%^20^. Open reading frames of coding regions were identified using TransDecoder (https://github.com/TransDecoder/TransDecoder) and functional annotation of the resulting transcripts was performed using BLAST^21^ against the NCBI NR database and predicted pathways were identified using InterProScan^22^.

The focus of this study was to develop reference transcriptomes for each component of this unique “food chain” (Fig. 1a). Although results for members of the *Dinophysis* food chain have been reported previously^11–13^, our focus was on two new isolates of *Dinophysis* (*D. acuminata* from the Chesapeake Bay, *D. ovum* from the Gulf of Mexico) and additional strains of *Mesodinium rubrum* and *Teleaulax amphioxeia* (Table 1). The use of multiple strains of a single harmful algal species has been recommended to address the physiological variability within a species^14^. Using the bioinformatics tools illustrated in Fig. 2, a total of 112,955 transcripts were identified for *D. acuminata*, 198,405 for *D. ovum*, 64,115 for *M. rubrum*-DK2009, 75,531 for *M. rubrum*-JAMR, and 154,041 for *T. amphioxeia* (Tables 2 and 3). The different sequencing depth between *D. acuminata and D. ovum* may explain the larger number of transcripts discovered for *D. ovum*. A reciprocal BLAST between the two *Dinophysis* species and clustering at 95% similarity yielded a total of 85,968 shared transcripts (Fig. 1b). The number of transcripts shared between the prey item *M. rubrum*-DK2009 and *D. acuminata* was 350 compared to 6,759 with *D. ovum* (Fig. 1b). These low numbers were expected because cultures of *Dinophysis* were extracted for analysis after all prey were depleted. Additionally, the number of transcripts shared between *M. rubrum*-JAMR and *D. acuminata* was 5,221 compared to 7,503 with *D. ovum*. A total of 54,540 transcripts were shared between *M. rubrum-*DK2009 and its prey, *T. amphioxeia* (Fig. 1b), and 49,297 between *M. rubrum-*JAMR and *T. amphioxeia*. The number of shared transcripts between the two *M. rubrum* strains DK2009 and JAMR was 43,115.

**Table 1:** Identification and isolation information for the *Dinophysis*, *Mesodinium*, and *Teleaulax* strains used in this study. All were grown at 18 °C, L1 medium^15^ at salinity of 22 ppt, and 100 µmol quant m^−2^ s^−1^. Raw read data are deposited in the NCBI BioProject PRJNA880267, Sequence Read Archive (SRA)^23–27^ and the Transcriptome Shotgun Assembly (TSA) at DDBJ/ENA/GenBank^28–32^. Annotated transcript datasets are deposited in Zenodo^33–37^.

Species	Strain	Collection Site	Collection Date	Isolator	SRA	TSA	Annotation
***Dinophysis acuminata***	DAVA01	Chesapeake Bay, Virginia, USA	March 2017	J. L. Smith	SRR21545757^23^	GKBP00000000^28^	zenodo.7325007^33^
***Dinophysis ovum***	DoSS3195	Surfside Beach, Texas, USA	March 2019	J. M. Fiorendino	SRR21545756^24^	GKBT00000000^29^	zenodo.7324981^34^
***Mesodinium rubrum***	MBL-DK2009	Helsingør Harbor, Denmark	2009	P. J. Hansen	SRR21545755^25^	GKBR00000000^30^	zenodo.7325017^35^
***Mesodinium rubrum***	JAMR	Inokushi Bay, Japan	2007	G. Nishitani	SRR21545753^26^	GKBQ00000000^31^	zenodo.7325034^36^
***Teleaulax amphioxeia***	K-0434	The Sound, Denmark	March 1990	D. Hill	SRR21545754^27^	GKBS00000000^32^	zenodo.7325044^37^

**Table 2:** Summary of RNA-seq results and number of reads after quality trimming, after removal of non-mRNA, and the final sequence reads used for assembly after normalization.

Species	Strain	Raw reads (2x)	SortMeRNA mRNA reads	Interleaved	Normalized reads
*Dinophysis acuminata*	DAVA01	109198428	52222701	32550934	16275467
*Dinophysis ovum*	DoSS3195	337941874	64102413	91332854	45666427
*Mesodinium rubrum*	MBL-DK2009	198141071	121606797	39315430	19657715
*Mesodinium rubrum*	JAMR	199950418	191740120	37202958	18601479
*Teleaulax amphioxeia*	K-0434	193206661	165206226	53502728	26751364

**Table 3:** Properties of the transcriptome assemblies.

Species	Strain	Trinity assembly	Velvet-Oases assembly	cd-hit-est-98 redundancy	TransDecoder CDS transcripts	% genes annotated	N50	BUSCO coverage
*Dinophysis acuminata*	DAVA01	237605	86414	225185	112955	78	747	60.4%
*Dinophysis ovum*	DoSS3195	420818	139676	401333	198405	77	867	81.2%
*Mesodinium rubrum*	DK2009	165470	78278	154413	64115	81	1056	76.8%
*Mesodinium rubrum*	JAMR	170381	95571	161523	75531	82	1056	79.7%
*Teleaulax amphioxeia*	K-0434	249599	102759	236984	154041	55	1395	87.9%

The assembled *de novo* transcriptomes for *D. acuminata* and *D. ovum* will serve as a reference for future experiments to assess the effect of different abiotic and biotic conditions and will also provide a useful resource for screening potential genes of interest to differentiate among the closely related species within the *D*. cf. *acuminata*-complex. The generated *de novo* transcriptomes for this collection of mixotrophic organisms will be a valuable resource for further downstream bioinformatics applications, including validation of gene expression, quantitative RNA-Seq analysis and comparative transcriptomics among strains of these harmful algal bloom species^14^.

## Methods

### Cell culturing and collection

Cultures of the kleptoplastic, mixotrophic species of *Dinophysis, D. acuminata* and *D. ovum*, the prey ciliate *Mesodinium rubrum*, and its prey, the cryptophyte *Teleaulax amphioxeia* (Table 1), were grown following the method described in Fiorendino *et al*. (10). Briefly, cultures were grown in L1-Si seawater medium^15^ at a salinity of 22, 18 °C, and under 100 µmol quanta m^−2^ s^−1^ on a 14: 10 light: dark cycle. Cultures were harvested by centrifugation at 3000 g for 15 mins. The cryptophyte *T. amphioxeia* was harvested at mid-exponential stage (~day 6). The *M. rubrum* and *Dinophysis* cultures were fed their respective prey at a 1:10 (predator: prey) ratio and harvested after the complete consumption of their cryptophyte or ciliate prey, respectively.

### RNA Extraction and sequencing

Total RNA was extracted from cell pellets using Extracta Plus RNA (QuantaBio, USA). Total RNA extraction was performed following the manufacturer′s guide. RNA concentration was measured using a Qubit RNA HS Assay kit (ThermoFisher Scientific, USA), and RNA integrity was evaluated using Agilent Fragment analyzer system (Agilent, USA).

Poly-A selected RNA libraries were prepared using the NEXTFLEX Rapid Directional RNA-seq kit 2.0 (Perkin Elmer, Waltham, MA) as per the manufacturer′s instructions. Each library was prepared with a unique barcode and pooled at equimolar concentrations. The pooled samples were sequenced on an Illumina NextSeq. 500 (Illumina, San Diego, CA) at a read length of 2 × 150 bp, targeting 60 million read pairs per sample.

### ***De novo*** assembly and gene annotation

High quality RNA-Seq reads (sequences) were used to generate the *de novo* transcriptome assemblies using the bioinformatics tools illustrated in Fig. 2. Raw sequence reads in fastq format were processed to remove adapters, poly-N (⩾10% read length), low-quality bases (Phred score < 10) and the last 10 bases were trimmed using the bbduk function in BBMap tool v. 38.90 (https://sourceforge.net/projects/bbmap/). Reads shorter than 125 bp were also discarded. Forward and reverse reads were concatenated using the bbrepair function. Non-mRNA reads were removed using SortMeRNA v. 4.3.4 with rRNA databases as reference^16^. The mRNA reads were normalized for depth based on kmer counts using the BBNorm function. Summary statistics for the number of total reads before and after precleaning are presented in Table 2. *De novo* transcriptomes were generated using Trinity v. 2.12.0^17^ with default settings and Velvet-master v. 1.2.10^18^-Oases-master v. 0.2.09^19^ with default settings, except for minimum length criterion set as 300 bp for the shortest transcripts. Both *de novo* transcriptomes were merged using cd-hit-est v. 4.8.1^20^ to reduce the transcript redundancy by 98% similarity and generate unique gene clusters. TransDecoder (https://github.com/TransDecoder/TransDecoder) was used to identify coding regions (ORF) of the assembled transcripts. The generated *de novo* assemblies were functionally annotated using the NCBI non-reductant protein database (NR) using BLAST tool v. 2.110^21^. InterProScan v. 5.55-88.0^22^ was used to identify potential proteins in pathways using the Pfam, PANTHER, Gene3D, SUPERFAMILY, TIGRFAM, HAMAP, SFLD, PRINTS datasets.

## Data Records

Three datasets were generated during the study. The first dataset consists of RNA-Seq raw reads from *D. acuminata* (DAVA01)^23^, *D. ovum* (DoSS3195)^24^, *M. rubrum* (DK2009)^25^, and (JAMR)^26^ and *T. amphioxeia* (K-0434)^27^, which were deposited in the NCBI Sequence Read Archive database (https://www.ncbi.nlm.nih.gov/bioproject/) under project identification number PRJNA880267 (Table 1). The second dataset contains the transcriptome assemblies for each of the five organisms which were deposited in the NCBI Transcriptome Shotgun Assembly (https://www.ncbi.nlm.nih.gov/genbank/tsa/) (Table 1)^28–32^. The third data set includes the annotated files that were deposited in Zenodo (Table 1)^33–37^ as XML files (Type 5 format of BLAST output). Headings in the Zenodo files include query sequence, query length, statistics for BLASTp, reference sequence and alignment.

## Technical Validation

After the initial FastQC check and precleaning steps, we assembled the *de novo* transcriptome assemblies with Trinity^17^ and Velvet-Oases^18,19^ (Table 3). We found that Trinity and Velvet-Oases produced different numbers of transcripts. The number of transcripts generated by Trinity was twice the number of transcripts from Velvet-Oases. The Trinity-Velvet-Oases merged strategy resulted in longer transcripts. Transcriptome assembly validation was done using Benchmarking Universal Single-Copy Orthologs (BUSCO) v. 4.1.4^38^. BUSCO core genes provide a qualitative estimate of the *de novo* transcriptome quality and completeness based on the evolutionarily informed expectation of the gene content from the near-universally conserved eukaryotic protein database (eukaryote_odb90). All five *de novo* transcriptome assemblies indicated high-quality assemblies with BUSCO coverage of 60–89% (Table 3). The CoDing sequences (CDS) obtained using TransDecoder revealed the highest number of genes in *D. ovum* (DoSS3195) while *M. rubrum* (DK2009) had the lowest number of genes (Table 3). N50 statistics appropriate for the *de novo* transcriptome assemblies were generated using the Trinity accessory scripts (Table 3). Functional annotation for these genes was performed using BLASTp with the maximum 3 best hits per gene and an e-value cutoff of 1e-20. The number of annotated genes ranged from 55–82% of the total transcripts (Table 3).

Using the bioinformatics tools illustrated in Fig. 2, the total number of transcripts for *D. ovum* exceeded the number for *D. acuminata;* this was probably due to the greater sequencing depth for *D. ovum* (Table 2). Note that although the number of transcripts in this analysis exceeded a previous report for *M. rubum*^12^, likely because of the increased depth of sequencing here, it is less than the number of transcripts identified by others^13^. To determine the number of transcripts shared between the two *Dinophysis* species, a reciprocal BLAST was performed and results clustered at 95% similarity (Fig. 1b).

## Data Availability

No custom code was generated.

## References

[1] Reguera B, Velo-Suárez L, Raine R, Park MG (2012). Harmful *Dinophysis* species: A review. Harmful Algae.

[2] Zingone, A.; Larsen, J. (Eds). Dinophysiales, in IOC-UNESCO Taxonomic Reference List of Harmful Micro Algae. https://www.marinespecies.org/hab (2022).

[3] Mitra A (2016). Defining planktonic protist functional groups on mechanisms for energy and nutrient acquisition: Incorporation of diverse mixotrophic strategies. Protist.

[4] Campbell L (2010). First harmful *Dinophysis* (DINOPHYCEAE, DINOPHYSIALES) bloom in the US is revealed by automated imaging flow cytometry. J. Phycol..

[5] Deeds JR, Wiles K, Heideman GB, White KD, Abraham A (2010). First US report of shellfish harvesting closures due to confirmed okadaic acid in Texas Gulf coast oysters. Toxicon.

[6] Trainer VL (2013). Diarrhetic shellfish toxins and other lipophilic toxins of human health concern in Washington state. Mar. Drugs.

[7] Tong MM (2015). Characterization and comparison of toxin-producing isolates of *Dinophysis acuminata* from New England and Canada. J. Phycol..

[8] Deeds JR (2020). Dihydrodinophysistoxin-1 produced by *Dinophysis norvegica* in the Gulf of Maine, USA and its accumulation in shellfish. Toxins.

[9] Wolny JL (2020). Characterization of *Dinophysis* spp. (Dinophyceae, Dinophysiales) from the mid-Atlantic region of the United States. J. Phycol..

[10] Fiorendino JM, Smith JL, Campbell L (2020). Growth response of *Dinophysis, Mesodinium*, and *Teleaulax* cultures to temperature, irradiance, and salinity. Harmful Algae.

[11] Hattenrath-Lehmann TK (2021). Transcriptomic and isotopic data reveal central role of ammonium in facilitating the growth of the mixotrophic dinoflagellate, *Dinophysis acuminata*. Harmful Algae.

[12] Altenburger A (2021). Limits to the cellular control of sequestered cryptophyte prey in the marine ciliate *Mesodinium rubrum*. ISMEJ.

[13] Lasek-Nesselquist E, Johnson MD (2019). A Phylogenomic approach to clarifying the relationship of Mesodinium within the Ciliophora: A case study in the complexity of Mixed-Species Transcriptome Analyses. Genome Biol. Evol..

[14] Wells ML (2015). Harmful algal blooms and climate change: Learning from the past and present to forecast the future. Harmful Algae.

[15] Guillard RRL, Hargraves PE (1993). *Stichochrysis immobilis* is a diatom, not a chrysophyte. Phycologia.

[16] Kopylova E, Noe L, Touzet H (2012). SortMeRNA: fast and accurate filtering of ribosomal RNAs in metatranscriptomic data. Bioinformatics.

[17] Grabherr MG (2011). Full-length transcriptome assembly from RNA-Seq data without a reference genome. Nat Biotech.

[18] Zerbino DR, Birney E (2008). Velvet: algorithms for de novo short read assembly using de Bruijn graphs. Genome Res.

[19] Schulz M, Zerbino D, Vingron M, Birney E (2012). Oases: Robust de novo RNA-seq assembly across the dynamic range of expression levels. Bioinformatics.

[20] Li WZ, Godzik A (2006). Cd-hit: a fast program for clustering and comparing large sets of protein or nucleotide sequences. Bioinformatics.

[21] Camacho C (2009). BLAST plus: architecture and applications. BMC Bioinformatics.

[22] Jones P (2014). InterProScan 5: genome-scale protein function classification. Bioinformatics.

[23] (2022). NCBI Sequence Read Archive.

[24] (2022). NCBI Sequence Read Archive.

[25] (2022). NCBI Sequence Read Archive.

[26] (2022). NCBI Sequence Read Archive.

[27] (2022). NCBI Sequence Read Archive.

[28] Gaonkar C, Campbell L (2022). GenBank.

[29] Gaonkar C, Campbell L (2022). GenBank.

[30] Gaonkar C, Campbell L (2022). GenBank.

[31] Gaonkar C, Campbell L (2022). GenBank.

[32] Gaonkar C, Campbell L (2022). GenBank.

[33] Gaonkar C, Campbell L (2022). Zenodo.

[34] Campbell L, Gaonkar C (2022). Zenodo.

[35] Campbell L, Gaonkar C (2022). Zenodo.

[36] Campbell L, Gaonkar C (2022). Zenodo.

[37] Campbell L, Gaonkar C (2022). Zenodo.

[38] Simao FA, Waterhouse RM, Ioannidis P, Kriventseva EV, Zdobnov EM (2015). BUSCO: assessing genome assembly and annotation completeness with single-copy orthologs. Bioinformatics.

[39] Anschutz AA, Flynn KJ, Mitra A (2022). Acquired phototrophy and its implications for bloom dynamics of the *Teleaulax*-*Mesodinium*-*Dinophysi*s-complex. Front. Mar. Sci..

